# ﻿Description of two species of the genus *Astrodia* Verrill, 1899 (Ophiuroidea, Euryalida, Asteronychidae), including a new species from seamounts in the West Pacific

**DOI:** 10.3897/zookeys.1123.87397

**Published:** 2022-10-04

**Authors:** Xiaojun Xie, Bo Lu, Jie Pang, Dongsheng Zhang

**Affiliations:** 1 Key Laboratory of Marine Ecosystem Dynamics, Second Institute of Oceanography, Ministry of Natural Resources, Hangzhou, China Second Institute of Oceanography, Ministry of Natural Resources Hangzhou China; 2 Southern Marine Science and Engineering Guangdong Laboratory (Zhuhai), Zhuhai, Guangdong, China Southern Marine Science and Engineering Guangdong Laboratory Zhuhai China; 3 School of Oceanography, Shanghai Jiao Tong University, Shanghai, China Shanghai Jiao Tong University Shanghai China

**Keywords:** Deep sea, molecular phylogeny, morphology, ophiuroids, taxonomy

## Abstract

Five specimens of Ophiuroidea from deep-sea seamounts in the West Pacific were collected and identified as two species, *Astrodiaduospina***sp. nov.** and *Astrodiaabyssicola*. The new species, *Astrodiaduospina***sp. nov.**, can be distinguished from its congeners by having indistinct or underdeveloped oral papillae, relatively short genital slits, crescent-shaped lateral arm plates, and plate-shaped external ossicles on the aboral surface of the disc. One specimen was identified as *Astrodiaabyssicola*, which has been reported in the north-western Pacific and the north-eastern coast of Japan. The most recent tabular key of *Astrodia* was revised with two more key characteristics added, the shape and presence of oral papillae and the number of arm spines. The phylogenetic relationship of *Astrodia* and *Asteronyx* was analyzed based on 16S and COI sequences. The discovery of the two species further expanded the geographical distribution of the genus *Astrodia*.

## ﻿Introduction

Class Ophiuroidea, as the largest group among echinoderms, with 2126 valid species (Stöhr et al. 2022), are widely distributed from the tropics to polar seas, and from the intertidal to the deep ocean. The Indo-Pacific, North Pacific, and South Pacific regions are reported to have relatively high ophiuroid species richness (Stöhr et al. 2012). Due to the technical limitations of deep sea exploration, the deep-sea ophiuroid fauna remains poorly known (Rodrigues et al. 2011). Seamounts are often of volcanic origin, with elevated topography from the deep-sea floor, which alters the flow of ocean currents and provides highly heterogeneous habitats serving as “hotspots” for deep-sea animals, especially for suspension-feeding epibenthic organisms (e.g. corals, sponges, and ophiuroids) (Yesson et al. 2011). Understanding the biodiversity of ophiuroids from seamounts will provide key information for the protection of this vulnerable ecosystem.

The order Euryalida Lamarck, 1816 comprises about 200 species from three families, Euryalidae Gray, 1840, Asteronychidae Ljungman, 1867, and Gorgonocephalidae Ljungman, 1867 (Stöhr et al. 2022). Among these, Asteronychidae is the smallest family with only 12 extant species from four genera (*Asteronyx* Müller & Troschel, 1842, *Astrodia* Verrill, 1899, *Astronebris* Downey, 1967 and *Ophioschiza* H.L. Clark, 1911). The genus *Astrodia* was erected by Verrill, in 1899 and currently comprises four species, *Astrodiaabyssicola* (Lyman, 1879), *Astrodiaexcavata* (Lütken & Mortensen, 1899), *Astrodiaplana* (Lütken & Mortensen, 1899) and *Astrodiatenuispina* (Verrill, 1884). *Astrodiatenuispina* was first described by Verrill (1884) under the name *Asteronyxtenuispina*, and was transferred to *Astrodia* by Verrill (1899). Koehler (1922) described a new species, *Astrodiabispinosa*, which was later regarded as a junior synonym of *Astrodiatenuispina* (Baker 1980). The most recent description of *Astrodiaplana* was published by Döderlein (1927). Recently, Okanishi and Fujita (2014) reviewed this genus and transferred *Ophiocreasabyssicola* Lyman, 1879 to *Astrodia*. In their review, Okanishi and Fujita (2014) provided interspecific distinguishing characteristics including the shape and arrangements of external ossicles on the aboral surface of the disc, length of genital slits in relation to the height of the disc, the shape of the lateral arm plates, presence or absence of a projection of the lateral arm plates on the middle to the distal portion of the arms. Additionally, the geographical distribution of the four species was summarized (Okanishi and Fujita 2014).

In this study, we describe a new species, *Astrodiaduospina* sp. nov., and redescribe *Astrodiaabyssicola*, from seamounts of the West Pacific. New interspecific diagnostic characteristics were identified, and the tabular key of Okanishi and Fujita (2014) for the genus *Astrodia* was updated. DNA sequences were used to infer the phylogenetic relationship of the two species with their congeners.

## ﻿Materials and methods

### ﻿Sample collection

**Figure 1.**
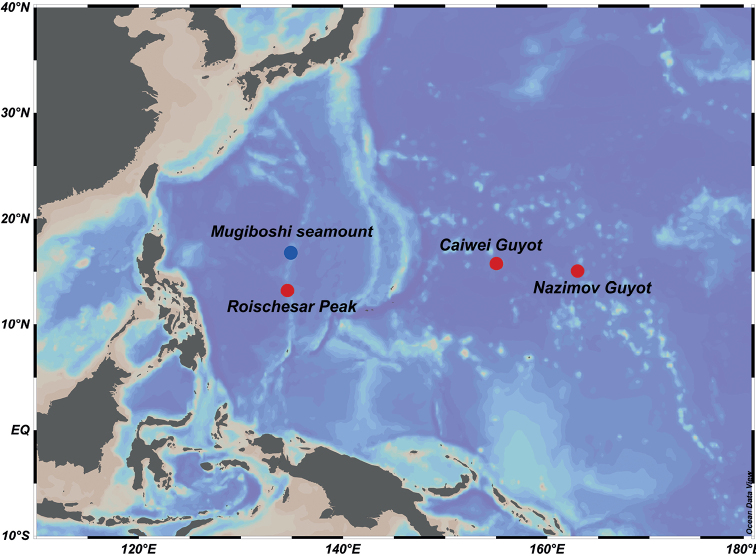
Sampling sites of two species in the Philippine Sea and the Northwest Pacific (red circles represent the sampling sites of *Astrodiaduospina* sp. nov., the blue circle represents the sampling site of *Astrodiaabyssicola*).

Five specimens of *Astrodia* were collected by ROV *HAILONG III*, ROV *HAILONG IV*, and HOV *JIAOLONG*, from seamounts in the Philippine Sea and the Northwest Pacific, during several COMRA’s cruises in 2013, 2020, and 2021 (Fig. 1). All specimens were preserved in 95% ethanol on board the vessels and photographed using a digital camera (Canon EOS 5D), then deposited in the repository of the Second Institute of Oceanography, Hangzhou, China (RSIO).

### ﻿Morphological analysis

Morphological characters were examined and photographed using a stereoscopic microscope (Zeiss Axio Zoom V16). Arm skeletons were examined with a Hitachi TM1000 scanning electron microscope. Skeletal elements were prepared by submerging in commercial bleach (2.5% NaOCl). Washed in distilled water and ethanol, air-dried, and mounted on a stub using dissolved carbon tapes. The following literature was used as references for the morphological analysis: Okanishi and Fujita (2014), Okanishi et al. (2018), Manso (2010), Baker (1980), and Martynov (2019).

### ﻿Molecular analysis

Several arm segments were dissected from each individual for genomic DNA extraction using DNeasy Blood & Tissue Kit (QIAGEN) following the manufacturer’s protocols. The COI sequences and 16S rRNA sequences were amplified with primers listed in Table 1. The PCR procedures were as follows: an initial denaturation step at 95 °C for 4 min followed by 35 cycles of 94 °C for 15 s, 50 °C for 30 s, and 72 °C for 1 min, and a final extension step at 72 °C for 10 min, for COI; an initial denaturation at 95 °C for 4 min, followed by 35 cycles of 94 °C for 15 s, 50 °C for 30 s, and 72 °C for 30 s, and a final extension at 72 °C for 7 min, for 16S. PCR reactions were performed using 25 µL volumes containing: 1 µL of DNA template, 1 µL of each primer, 9.5 µL of dd H_2_O, and 12.5 µL of 2 × Phanta Max Master Mix (Vazyme, China). PCR products were purified with a QIAquick PCR purification kit (QIAGEN) following the protocol supplied by the manufacturer. Sequencing was performed by Sangon Biotech (Shanghai, China) on an ABI 3730XL DNA analyzer (Applied Biosystems, Foster City, CA, USA). Forward and reverse sequences were de novo assembled and edited using Geneious Prime 2021 (https://www.geneious.com), deposited in GenBank (COI: OP328780–OP328783; 16S: OP325290–OP325293).

**Table 1.** Information on primers used for PCR programs.

Primer	Sequence
Oph-COI-F	TTTCAACTAATCAYAAGGAYATWGG
Oph-COI-R	CTTCAGGRTGWCCRAARAAYCA
16Sar	CGCCTGTTTATCAAAAACAT
16Sbr	CCGGTCTGAACTCAGATCACGT

Seventy-two 16S sequences and 28 COI sequences of Asteronychidae were downloaded from the NCBI. In total, 78 16S sequences and 34 COI sequences (Table 2), including four new 16S sequences and four new COI sequences were used for phylogenetic analysis, with two species of *Asteroschema* as the outgroup. COI and 16S were aligned using Clustal Omega (Sievers and Higgins 2014) as a plug-in in Geneious with default settings, respectively. Maximum likelihood trees were inferred based on a concatenated alignment of 16S and COI, as well as an alignment of 16S and COI respectively. IQ-TREE was used to perform the maximum likelihood bootstrap method (http://iqtree.cibiv.univie.ac.at/) (Nguyen et al. 2015), with the substitution model GTR+I+G, bootstrap support values determined by the ultrafast bootstrap algorithm for 100,000 replicates (Hoang et al. 2018). The best substitution model was selected by ModelFinder as a plug-in in IQ-TREE websites. (Kalyaanamoorthy et al. 2017).

**Table 2.** Voucher specimens and accession numbers of COI and 16S sequence data used in the phylogenetic analysis (IDSSE, Institute of Deep-sea Science and Engineering, China; MV, Museums Victoria, Australia; NSMT, National Museum of Nature and Science, Japan; RSIO, Second Institute of Oceanology, China; SIO, Scripps Institution of Oceanography, USA).

Species	Locality	Voucher number	CO1	16S	Code from Okanishi et al. (2018)
*Asteronyxlongifissus*	Monterey, California	SIO: BIC: E6108	-	KM014337	-
*Asteronyxloveni*	South China Sea	IDSSE-EEB-SW0002	MZ198756	MZ203264	-
*Asteronyxloveni*	New Zealand	MV F188855	KU895061	-	-
*Asteronyxloveni*	Off Abashiri, Hokkaido	NSMT E-6904-A	-	LC276316	OK-226
*Asteronyxloveni*	Off Abashiri, Hokkaido	NSMT E-6904-B	-	LC276354	OK-315
*Asteronyxloveni*	Off Abashiri, Hokkaido	NSMT E-6904-C	LC276289	LC276330	OK-256
*Asteronyxloveni*	Off Abashiri, Hokkaido	NSMT E-6904-G	LC276290	LC276331	OK-257
*Asteronyxloveni*	Off Abashiri, Hokkaido	NSMT E-6904-H	LC276282	LC276317	OK-227
*Asteronyxloveni*	Off Abashiri, Hokkaido	NSMT E-6904-I	-	LC276359	OK-339
*Asteronyxloveni*	Off Abashiri, Hokkaido	NSMT E-6904-J	-	LC276350	OK-295
*Asteronyxloveni*	Off Abashiri, Hokkaido	NSMT E-6904-K	-	LC276332	OK-258
*Asteronyxloveni*	Off Abashiri, Hokkaido	NSMT E-6904-L	-	LC276358	OK-337
*Asteronyxloveni*	Off Abashiri, Hokkaido	NSMT E-6904-R	-	LC276334	OK-262
*Asteronyxloveni*	Off Abashiri, Hokkaido	NSMT E-6904-S	-	LC276353	OK-314
*Asteronyxloveni*	Off Abashiri, Hokkaido	NSMT E-6904-T	-	LC276333	OK-261
*Asteronyxloveni*	Off Abashiri, Hokkaido	NSMT E-6951-B	-	LC276343	OK-281
*Asteronyxloveni*	Off Abashiri, Hokkaido	NSMT E-6951-C	LC276292	LC276337	OK-269
*Asteronyxloveni*	Off Abashiri, Hokkaido	NSMT E-6951-D	-	LC276344	OK-284
*Asteronyxloveni*	Off Abashiri, Hokkaido	NSMT E-6951-F	-	LC276336	OK-268
*Asteronyxloveni*	Off Abashiri, Hokkaido	NSMT E-6951-G	-	LC276341	OK-279
*Asteronyxloveni*	Off Abashiri, Hokkaido	NSMT E-6951-H	LC276291	LC276335	OK-267
*Asteronyxloveni*	Off Miyako, Iwate	NSMT E-6943-A	LC276288	LC276329	PT-253
*Asteronyxloveni*	Off Miyako, Iwate	NSMT E-6256	AB758757	AB605076	PT-41
*Asteronyxloveni*	Off Miyako, Iwate	NSMT E-5641-A	LC276284	LC276320	PT-238
*Asteronyxloveni*	Off Miyako, Iwate	NSMT E-5641-B	LC276285	LC276321	PT-239
*Asteronyxloveni*	Off Miyako, Iwate	NSMT E-5641-C	-	LC276322	PT-240
*Asteronyxloveni*	Off Miyako, Iwate	NSMT E-5641-D	LC276286	LC276323	PT-241
*Asteronyxloveni*	Off Miyako, Iwate	NSMT E-5641-E	-	LC276324	PT-242
*Asteronyxloveni*	Off Miyako, Iwate	NSMT E-5638-A	LC276278	LC276308	PT-213
*Asteronyxloveni*	Off Miyako, Iwate	NSMT E-5638-B	-	LC276352	PT-306
*Asteronyxloveni*	Off Miyako, Iwate	NSMT E-5638-D	-	LC276357	PT-323
*Asteronyxloveni*	Off Miyako, Iwate	NSMT E-5638-E	-	LC276356	PT-320
*Asteronyxloveni*	Off Miyako, Iwate	NSMT E-5637-A	-	LC276310	PT-215
*Asteronyxloveni*	Off Miyako, Iwate	NSMT E-5637-B	LC276281	LC276314	PT-220
*Asteronyxloveni*	Shima Spur, Mie	NSMT E-6360	-	LC276302	PM-199
*Asteronyxloveni*	Shima Spur, Mie	NSMT E-6983	LC276280	LC276312	PM-218
*Asteronyxloveni*	Shima Spur, Mie	NSMT E-6983	-	LC276347	PM-290
*Asteronyxloveni*	Shima Spur, Mie	NSMT E-6982	-	LC276309	PM-214
*Asteronyxloveni*	Off Tosa, Kochi	NSMT E-1143-A	-	LC276318	PK-231
*Asteronyxloveni*	East China Sea, west of Japan	NSMT E-6986-A	LC276273	LC276298	ECS-195
*Asteronyxloveni*	East China Sea, west of Japan	NSMT E-6986-C	LC276272	LC276297	ECS-194
*Asteronyxreticulata*	East of Hiraji Bank, Nagasaki	NSMT E-6912	-	LC276355	ECS-316
*Asteronyxreticulata*	East of Hiraji Bank, Nagasaki	NSMT E-6915	-	LC276338	ECS-272
*Asteronyxreticulata*	East of Hiraji Bank, Nagasaki	NSMT E-7016	-	LC276301	ECS-198
*Asteronyxreticulata*	East of Naka-Kasayama Bank, Nagasaki	NSMT E-6908-C	-	LC276293	ECS-190
*Asteronyxreticulata*	East of Naka-Kasayama Bank, Nagasaki	NSMT E-6908-D	LC276271	LC276296	ECS-193
*Asteronyxreticulata*	East of Naka-Kasayama Bank	NSMT E-6931	LC276279	LC276311	ECS-217
*Asteronyxreticulata*	West of Gajajima Isl. Kagoshima	NSMT E-6354	-	LC276305	ECS-204
*Asteronyxreticulata*	East of Hiraji Bank, Nagasaki	NSMT E-6910	-	LC276300	ECS-197
*Asteronyxreticulata*	East of Hiraji Bank, Nagasaki	NSMT E-6911	-	LC276294	ECS-191
*Asteronyxreticulata*	East of Hiraji Bank, Kagoshima	NSMT E-6926	-	LC276342	ECS-280
*Asteronyxreticulata*	East of Hiraji Bank, Kagoshima	NSMT E-6929	-	LC276304	ECS-203
*Asteronyxreticulata*	West off Takarajima Isl.	NSMT E-6355	LC276274	LC276299	ECS-196
*Asteronyxreticulata*	West of Amami Ohshima Isl., Kagoshima	NSMT E-6351-A	-	LC276325	ECS-243
*Asteronyxreticulata*	West of Amami Ohshima Isl., Kagoshima	NSMT E-6942-B	-	LC276339	ECS-274
*Asteronyxreticulata*	West of Ensei Knoll, Kagoshima	NSMT E-6921	-	LC276349	ECS-294
*Asteronyxreticulata*	West of Ensei Knoll, Kagoshima	NSMT E-6922-A	LC276287	LC276328	ECS-249
*Asteronyxreticulata*	West of Ensei Knoll, Kagoshima	NSMT E-6925-A	-	LC276326	ECS-247
*Asteronyxreticulata*	West of Ensei Knoll, Kagoshima	NSMT E-6925-B	-	LC276327	ECS-248
*Asteronyxreticulata*	East China Sea, west of Japan	NSMT E-7001	LC276276	LC276306	ECS-205
*Asteronyxreticulata*	East China Sea, west of Japan	NSMT E-7002	LC276277	LC276307	ECS-206
*Asteronyxreticulata*	Off Amami Ohshima Isl. Kagoshima	NSMT E-6352	-	LC276315	ECS-223
*Asteronyxreticulata*	West of Minami-Ensei Knoll, Kagoshima	NSMT E-6916	-	LC276345	ECS-286
*Asteronyxreticulata*	West of Minami-Ensei Knoll, Kagoshima	NSMT E-6923-A	-	LC276340	ECS-278
*Asteronyxreticulata*	West of Minami-Ensei Knoll, Kagoshima	NSMT E-6923-B	LC276275	LC276303	ECS-202
*Asteronyxreticulata*	East China Sea, west of Japan	NSMT E-7003-A	-	LC276351	ECS-303
*Asteronyxreticulata*	East China Sea, west of Japan	NSMT E-7003-B	-	LC276346	ECS-288
*Asteronyxreticulata*	East China Sea, west of Japan	NSMT E-7000-A	-	LC276348	ECS-291
*Asteronyxreticulata*	West of Minami-Ensei Knoll, Kagoshima	NSMT E-6920	LC276270	LC276295	ECS-192
*Asteronyxreticulata*	Off Iejima Isl., Okinawa	NSMT E-6987	-	LC276313	ECS-219
*Asteronyx* sp.	Between Yakushima Isl and Tanegashima Isl., Kagoshima	NSMT E-3157-B	LC276283	LC276319	PSW-237
*Asteronyxluzonicus*	South China Sea	IDSSE-EEB-SW0003	MZ198757	MZ203265	-
*Astrodiaabyssicola*	Miyagi, off Onahama	NSMT E-6257	AB758828	AB605077	-
*Astrodiaabyssicola*	Philippine Sea, KPR Seamount	RSIO68002	OP328783	OP325293	-
*Astrodiaduospina* sp. nov.	Philippine Sea, KPR Seamount	RSIO59012	OP328780	OP325290	-
*Astrodiaduospina* sp. nov.	Northwest Pacific, Ko-Hakucho-Guyout Seamount	RSIO61068	OP328781	OP325291	-
*Astrodiaduospina* sp. nov.	Northwest Pacific, RB Seamount	RSIO61069	OP328782	OP325292	-
*Asteroschemaajax*	Off Lord Howe Isl.	MV F99759	AB758762	AB605078	-
*Asteroschemaclavigerum*	North Atlantic	haplotype 1	HM587850	HM587828	-

## ﻿Results and discussion

### ﻿Systematics

**Class Ophiuroidea Gray, 1840**

**Order Euryalida Lamarck, 1816**

**Family Asteronychidae Ljungman, 1867**

#### Genus *Astrodia* Verrill, 1899

##### ﻿ Astrodia duospina sp. nov.

Taxon classificationAnimaliaEuryalidaAsteronychidae

E3D170A2-8236-54E3-9FF4-42C3EB3D5CB6

https://zoobank.org/FC14B3BB-E9BB-4E61-A959-266A0CA733C8

**Figure 2.**
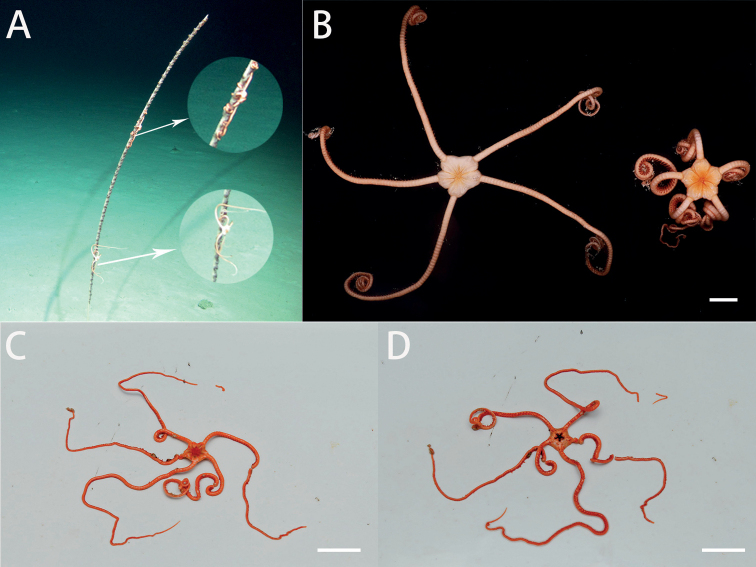
In situ and on-board photos of *Astrodiaduospina* sp. nov. **A** photo in situ (RSIO61068: the individual below, RSIO61069: the individual above, attached to an unidentified sea pen species) **B** photo on board (RSIO61068: the individual on the left, RSIO61069: the individual on the right) **C, D** photos on board (RSIO31004), aboral side (**C**), oral side (**D**). Scale bars: 10 mm (**B**); 20 mm (**C, D**).

**Figure 3.**
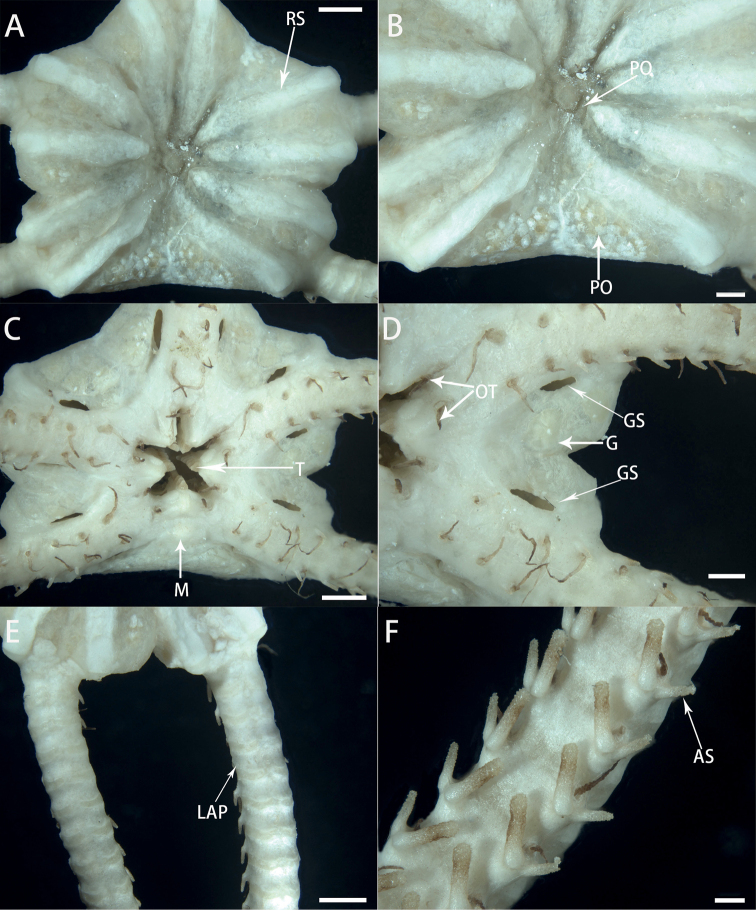
Morphological characters of *Astrodiaduospina* sp. nov. (holotype: RSIO61068) **A** aboral view of the disc **B** periphery of the aboral disc **C** oral view of the disc **D** genital silts **E** aboral view of the arms **F** arms spines. Abbreviations: **RS** radial shield; **PO** plate-shaped ossicle; **M** madreporite; **T** teeth; **OT** oral tentacle; **GS** genital slit; **G** gonad; **LAP** lateral arm plate; **AS** arm spine. Scale bars: 2 mm (**A, C, E**); 1 mm (**B, D**); 0.5 mm (**F**).

**Figure 4.**
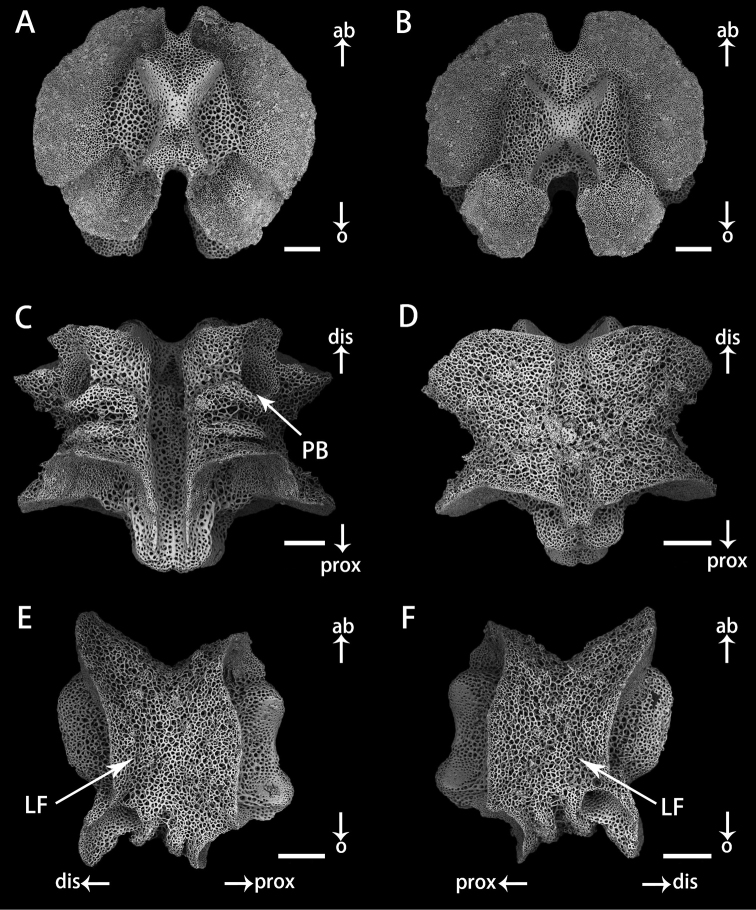
Vertebrae in basal arm of *Astrodiaduospina* sp. nov. (holotype: RSIO61068) **A** proximal view **B** distal view **C** oral view **D** aboral view **E, F** lateral view. Abbreviations: **PB** podial basin; **LF** lateral furrow. Scale bars: 200 μm (**A–F**).

**Figure 5.**
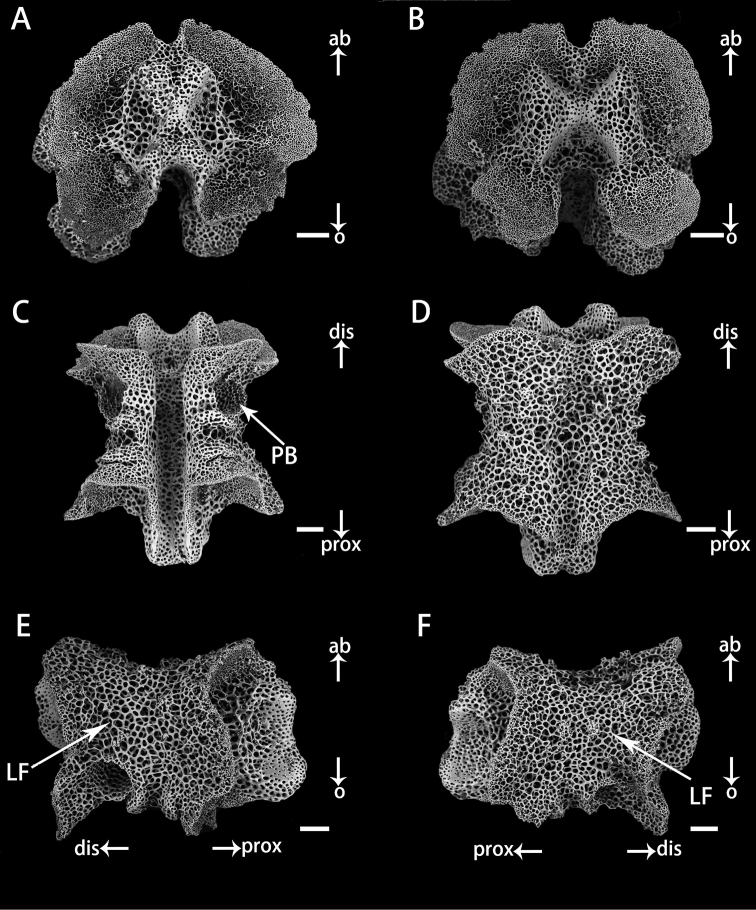
Vertebrae in distal arm of *Astrodiaduospina* sp. nov. (holotype: RSIO61068) **A** proximal view **B** distal view **C** oral view **D** aboral view **E, F** lateral view. Abbreviations: **PB** podial basin; **LF** lateral furrow. Scale bars: 100 μm (**A–F**).

**Figure 6.**
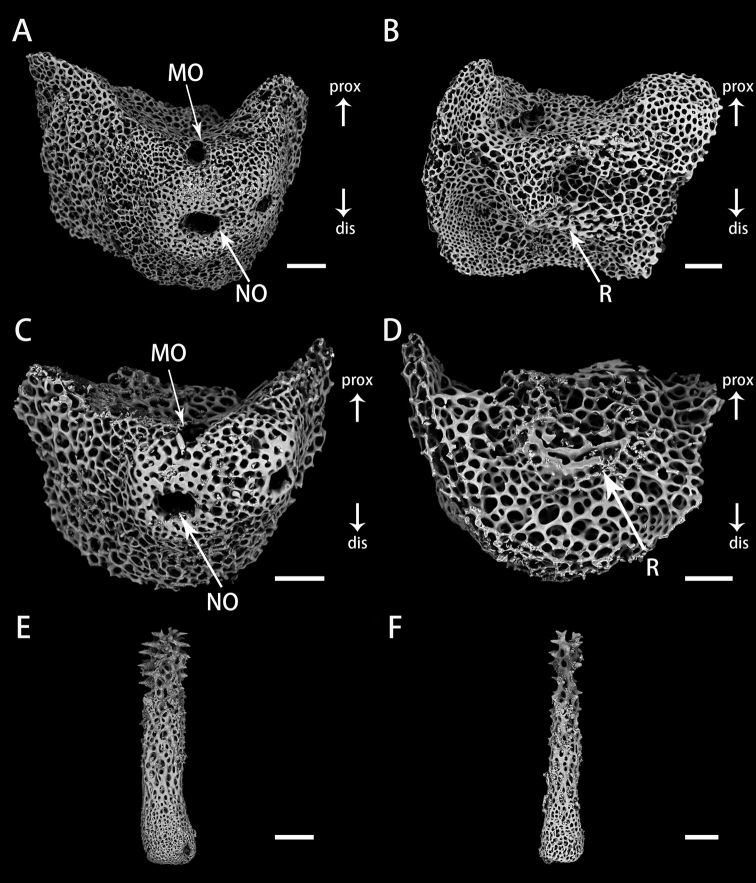
Lateral arm plates and arm spines of *Astrodiaduospina* sp. nov. (holotype: RSIO61068) **A, B** lateral arm plates from proximal arm, outer view (**A**), inner view (**B**) **C, D** lateral arm plates from distal arm, outer view (**C**), inner view (**D**) **E, F** arm spines from proximal (**E**) and distal arm (**F**). Abbreviations: **MO** muscle opening; **NO** nerve opening; **R** ridge. Scale bars: 200 μm (**E**); 100 μm (**F**); 90 μm (**A, B**); 60 μm (**C, D**).

**Figure 7.**
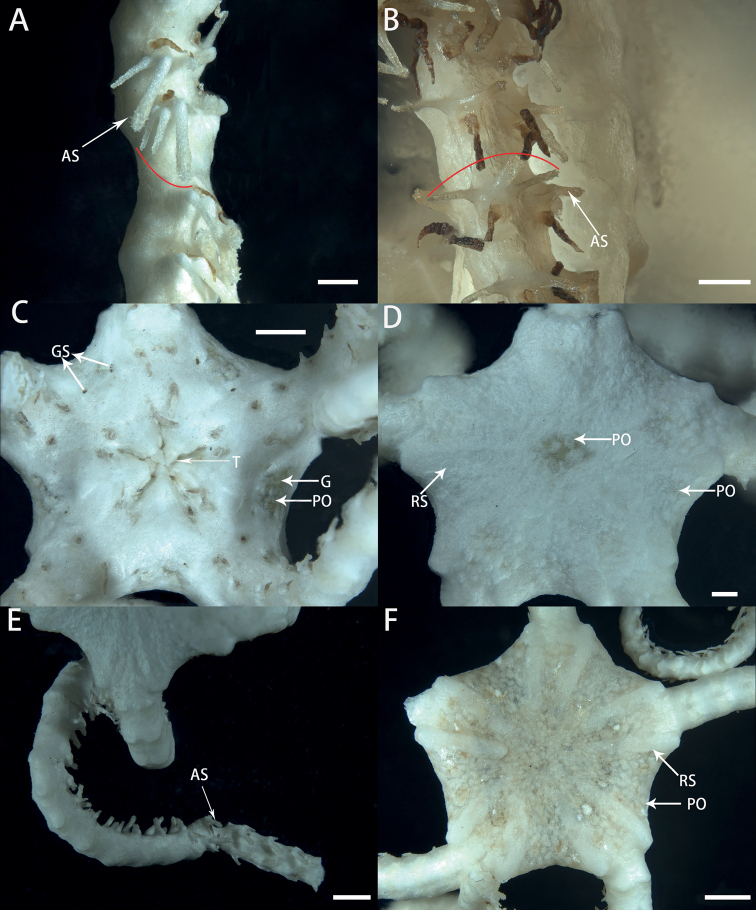
Morphological characters of paratypes of *Astrodiaduospina* sp. nov. **A** arm spines of RSIO31004 **B** arm spines of RSIO61069 **C–E** pictures of RSIO59012, oral disc (**C**), aboral disc (**D**), arm and arm spines (**E**) **F** aboral disc of RSIO31004. These specimens have exceptionally three arm spines for an arm segment (shown by an arc in A and B). Abbreviations: **AS** arm spine; **T** teeth; **PO** plate-shaped ossicle; **GS** genital slit; **G** gonad; **RS** radial shield. Scale bars: 2 mm (**F**); 1 mm (**C, E**); 0.5 mm (**A, B, D**).

Figs 2 , 3 , 4 , 5 , 6 , 7

###### Material examined.

***Holotype***: China • 1 specimen; Northwest Pacific, Nazimov Guyot; 15°11.34'N, 162°49.26'E; depth 2713 m; 16 September 2020; collected by ROV HAILONG III; preserved in alcohol; RSIO61068. ***Paratypes***: China • 1 specimen; Northwest Pacific, Nazimov Guyot; 15°11.34'N, 162°49.26'E; depth 2713 m; 16 September 2020; collected by ROV HAILONG III; preserved in alcohol; RSIO61069 • 1 specimen; Northwest Pacific, Caiwei Guyot; 15°40.61'N, 154°53.77'E; depth 2744 m; 7 September 2013; collected by HOV JIAOLONG; preserved in alcohol; RSIO31004 • 1 specimen; the Philippine Sea, Kyushu-Palau Ridge, Roischesar Peak; 13°20.85'N, 134°32.81'E; depth 1900–2000 m; 2 August 2020; collected by ROV HAILONG IV; preserved in alcohol; RSIO59012.

###### Diagnosis.

Disc raised high above the arm. Aboral disc with plate-shaped external ossicles in the center and on the periphery. Radial shield narrow, longer than wide. Teeth triangular, oral papillae indistinct or underdeveloped. Genital slits short, approximately one-fourth of the height of the disc. Lateral arm plates crescent and not projecting on arms. Arm spines no more than two.

###### Description of holotype.

Disc pentagonal, notched interradial edges, 14 mm in diameter, 4.7 mm in height. Aboral surface almost flat, slightly depressed in the center, entirely covered by thickened skin with plate-shaped external ossicles in the center, about 220 μm long (Fig. 3A). Peripheral disc covered with a few plate-shaped external ossicles, similar to those in the center but larger, approximately twice in length. Radial shields narrow, tumid, bar-like, without granules or spines, and almost reach center of disc (Fig. 3A, B). Approximately 7.2 mm long and 550 μm wide in the center and 1.1 mm wide at periphery.

Oral surface flat, covered by thickened skin. Oral shield small to invisible, one madreporite. Adoral shield obscured by skin (Fig. 3C). Oral interradial surface covered with several plate-shaped external ossicles (Fig. 3C). Six teeth, triangular, forming vertical row on dental plate, each jaw covered by a pair of conical oral tentacles (Fig. 3C). Oral papillae invisible or underdeveloped. Two genital slits, small, about 1/4 as long of disc height (1.3 mm long and 260 μm wide), present on oral side of each interradius (Fig. 3D). Gonads visible on each interradius (Fig. 3C, D).

Five arms, long and slender, about eight to nine times as long as disc diameter, no abrupt change in width basally (Fig. 3E). Proximal segments 2.5 mm wide and 1.7 mm high, with arched aboral surface and flattened oral surface (Fig. 3E), gradually tapering toward tip. Arm spines only present on ventral side. First to fourth tentacle pores with one arm spine and following tentacle pores with two arm spines. Outer arm spines slightly shorter than inner ones at proximal segments, but only three-fifths as long as inner spines on middle and distal segments (Fig. 3F).

***Color*.** Pink in situ, white in alcohol (Fig. 2).

***Ossiclemorphology of holotype*.** Vertebrae articulation streptospondylous, wider than long in proximal segments (Fig. 4A, B), longer than wide in distal segments (Fig. 5A, B). Oral side of each vertebra with longitudinal groove along midline, deeply depressed, and no oral bridge (Figs 4C, 5C). Pair of podial basins on oral side moderate in size (Figs 4C, 5C). Aboral side of each arm vertebra with longitudinal aboral groove, moderately depressed (Figs 4D, 5D). Lateral furrow of vertebrae declining obliquely from aboral to oral side (Figs 4E–F, 5E–F). Lateral arm plates crescent-shaped, each associated with one or two arm spines and spine articulations with nerve and muscle opening separated. Spine articulation bulges outward (Fig. 6A, C). A ridge on inner side of lateral arm plate, parallel to proximal edge (Fig. 6B, D). Arm spines cylindrical, never hooked, bearing fine thorns at tip throughout arms (Figs 3F, 6E–F).

###### Description of paratypes.

Two paratypes (RSIO31004, RSIO61069) share the same morphological characteristics as the holotype, disc diameter 10.17 and 13.94 mm, about 1/10 and 1/9 as wide as the length of the arms, respectively. However, the radial shields of RSIO31004 are shorter than the radial shields of the holotype and of

RSIO61069 (Fig. 7F). Three arm spines exceptionally occurred only once in both paratypes (RSIO31004 and RSIO61069), the innermost arm spine of RSIO61069 is the longest and the stoutest, while the middle arm spine of RSIO31004 is the stoutest. (Fig. 7A, B). The other paratype (RSIO59012) is smaller, only 6 mm in disc diameter, about 1/3 as wide as the length of the arms and may be a juvenile of this species. The radial shields and the genital silts are much shorter than in the other three specimens (Fig. 7C, D). Likewise, the arm spines are shorter than one segment (Fig. 7E)

###### Etymology.

The species name *duo* is derived from the Latin numeral word, meaning two, and Latin feminine noun, *spina*, meaning spine, referring to the presence of no more than two arm spines throughout the arm.

###### Remarks.

This new species falls within the genus *Astrodia* by only possessing cylindrical unhooked arm spines. The new species resembles *Astrodiaabyssicola* mostly by having plate-shaped external ossicles on the aboral disc and crescent-shaped lateral arm plates. However, the oral papillae are indistinct or underdeveloped in *Astrodiaduospina*, which can be used to distinguish the two species from each other (Fig. 3D). Moreover, the genital slits are very short in *Astrodiaabyssicola*, which are only one-fifth of the height of the disc, while *Astrodiaduospina* has larger genital slits, being longer than one-fourth the height of the disc (Fig. 3C). *Astrodiaduospina* can easily be distinguished from A.plana and A.excavata by external ossicles and lateral arm plates. External ossicles are plate-shaped on the aboral surface of the disc in *Astrodiaduospina* (Fig. 3A, B), but are absent in *A.plana*. Lateral arm plates are not projecting in the new species (Fig. 3E), but are distinctly projecting from the oral surface of the arm in *A.excavata*. Additionally, the new species differs from *A.tenuispina* by having distinctly smaller genital slits (Figs 3C, 7C).

*Astrodiatenuispina* is a widely distributed species and was characterized by having slender unhooked arm spines, small and short oral papillae, separated genital slits (Verrill 1884). Baker (1980) compared specimens from south of Australia and the northwest Atlantic, described this species with 2 or 3 arm spines, and imbricating punctate scales on the disc surface. Okanishi and Fujita (2014) redescribed this species as with plate-shaped external ossicles on the periphery of the aboral disc, granule-shaped on the central disc, genital slits half of the height of the disc, lateral arm plates not projecting. According to these descriptions, *A.duospina* sp. nov. can be differentiated from *A.tenuispina* by having smaller genital slits and indistinct oral papillae. Furthermore, in two of the three large specimens of the new species, three arm spines were observed exceptionally at one arm segment (Fig. 7A, B), while the other three species possess three arm spines at several successive segments in the middle part of the arms. Since only a small number of specimens were examined, this characteristic was not used to distinguish the new species from its congeners, and more specimens should be examined before a robust result can be achieved.

##### ﻿ Astrodia abyssicola

Taxon classificationAnimaliaEuryalidaAsteronychidae

(Lyman, 1879)

6318B1AF-001E-5BE3-A22D-1E19EC49DFBE

**Figure 8.**
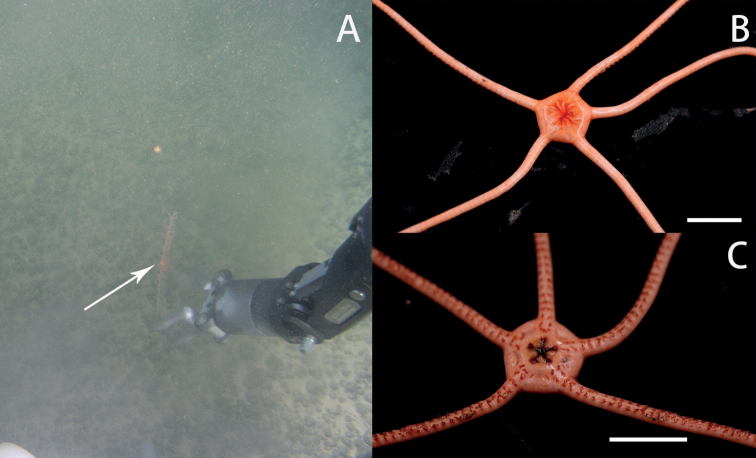
In situ and on-board photos of *Astrodiaabyssicola***A** photo in situ (RSIO68002, attached to an unidentified sea pen species) **B, C** photos on board (RSIO68002), aboral side (**B**), oral side (**C**). Scale bars: 10 mm (**B, C**).

**Figure 9.**
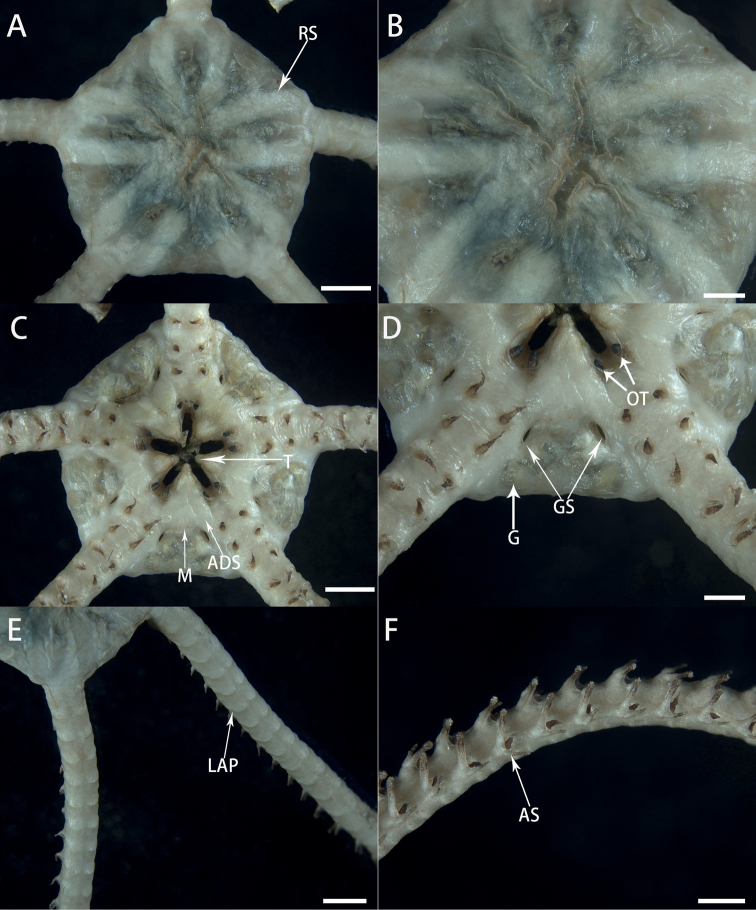
Morphological characters of *Astrodiaabyssicola* (RSIO68002) **A** aboral view of the disc **B** center of the aboral disc **C** oral view of the disc **D** genital silts **E** aboral view of the arms **F** arms spines. Abbreviations: **RS** radial shield; **M** madreporite; **T** teeth; **ADS** adoral shield; **OT** oral tentacle; **GS** genital slit; **G** gonad; **LAP** lateral arm plate; **AS** arm spine. Scale bars: 2 mm (**A, C, E**); 1 mm (**B, D, F**).

**Figure 10.**
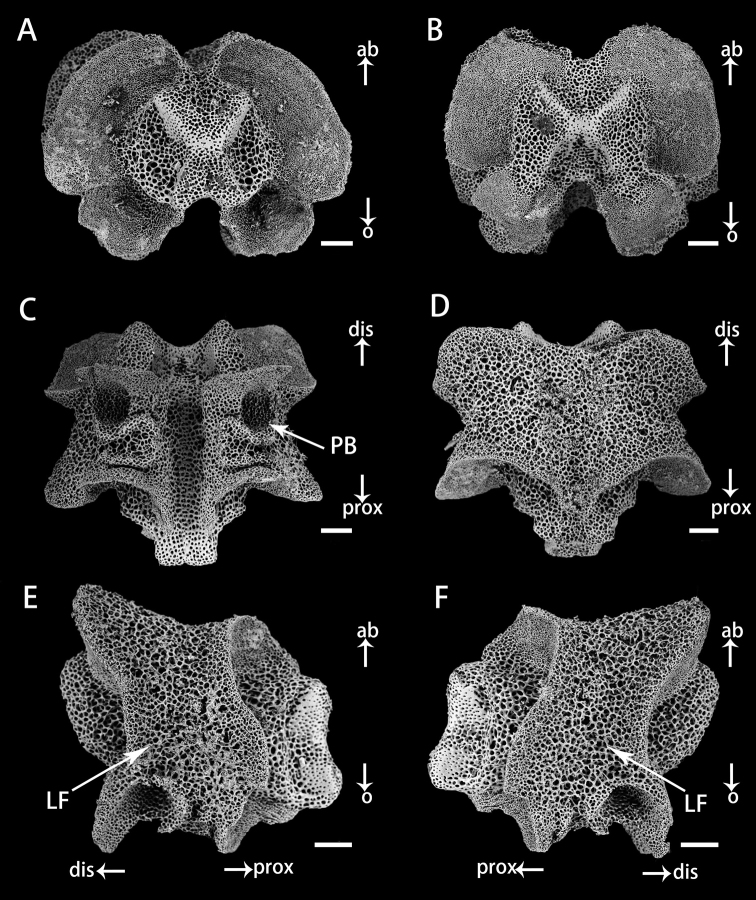
Vertebrae in basal arm of *Astrodiaabyssicola* (RSIO68002) **A** proximal view **B** distal view **C** oral view **D** aboral view **E, F** lateral view. Abbreviations: **PB** podial basin; **LF** lateral furrow. Scale bars: 150 μm (**A–F**).

**Figure 11.**
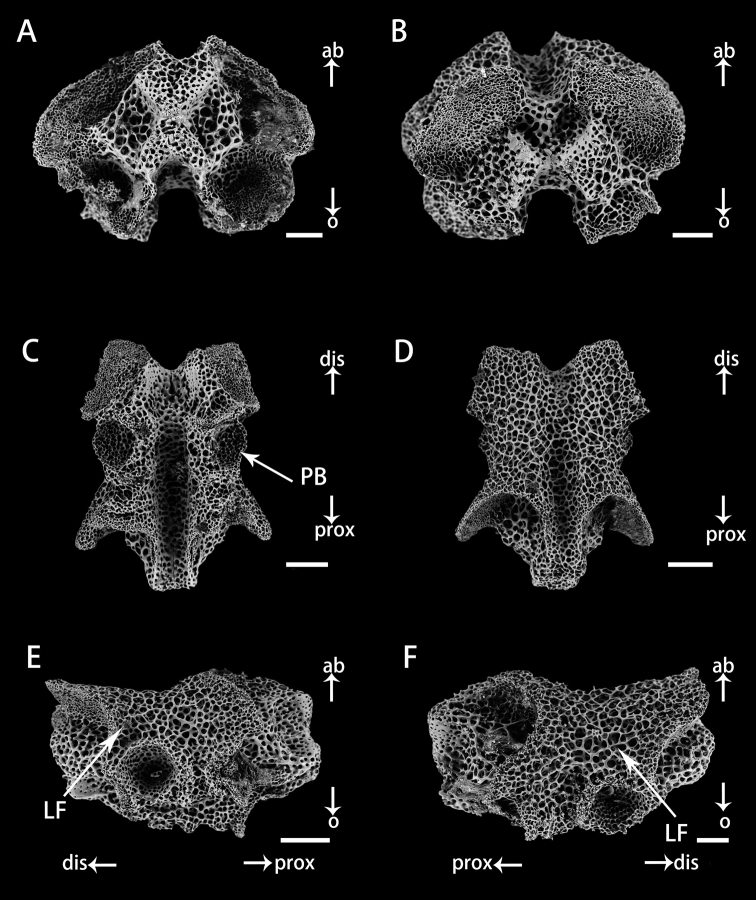
Vertebrae in distal arm of *Astrodiaabyssicola* holotype: RSIO68002) **A** proximal view **B** distal view **C** oral view **D** aboral view **E, F** lateral view. Abbreviations: **PB** podial basin; **LF** lateral furrow. Scale bars: 150 μm (**C, D, E**); 90 μm (**A, B, F**).

**Figure 12.**
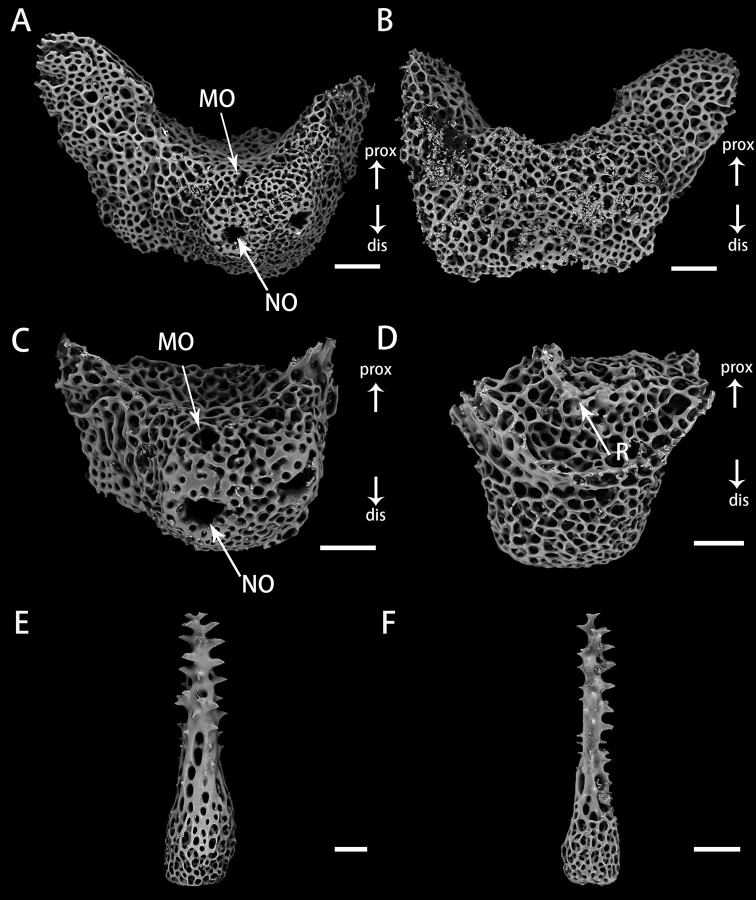
Lateral arm plates and arm spines of *Astrodiaabyssicola* (RSIO68002) **A, B** lateral arm plates from the proximal arm, outer view (**A**), and inner view (**B**) **C, D** lateral arm plates from the distal arm, outer view (**C**), and inner view (**D**) **E, F** arm spines from proximal (E) and distal (F). Abbreviations: **MO** muscle opening; **NO** nerve opening; **R** ridge. Scale bars: 90 μm (**A, B, F**); 60 μm (**C, D, E**).

Figs 8 , 9 , 10 , 11 , 12

Ophiocreas abyssicola Lyman, 1879: 64–65, plate 17, figs 470–473.
Astrodia abyssicola : Okanishi and Fujita 2014: 188–192, figs 2–4.

###### Material examined.

China • 1 specimen; Philippine Sea, Kyushu-Palau Ridge, Mugiboshi Seamount; 16.57.14'N, 134.52.7'E; depth 3225 m; 11 August 2021; collected by an HOV JIAOLONG; preserved in alcohol; RSIO68002.

###### Description.

Disc pentagonal and almost flat, 10 mm in diameter, 3.2 mm in height, skin wrinkled under dry conditions (Fig. 9A, B). Aboral surface of disc lacks external ossicles (Fig. 9A, B). Radial shields narrow, slightly tumid, bar-like, without granules or spines, and almost reaching center of disc. (Fig. 9A). Approximately 3.8 mm long and 0.6 mm wide in center and 0.8 mm wide on periphery (Fig. 9A).

Oral surface flat, covered by thin skin, and lacking external ossicles (Fig. 9C). Oral shield triangular, one madreporite (Fig. 9D). Adoral shield big and thick, quadrangular, and longer than wide (Fig. 9D). Teeth spearhead-shaped, vertically on dental plate; each jaw bears a pair of short, conical oral tentacles (Fig. 9C). Oral papillae indistinct or underdeveloped (Fig. 9C). Two genital slits very short, 560 μm long and 110 μm wide, present on oral side of each interradius. Gonads visible in each interradius (Fig. 9D).

Five arms, long and slender, about nine to ten times as long as disc diameter, no abrupt change in width basally (Fig. 9E). Proximal portion of arm 1.8 mm wide and 420 μm high, with arched aboral surface and flattened oral surface. Arms tapering gradually toward tip. Arm spines only present in ventral part of arm. First to third tentacle pores without arm spines, fourth tentacle pores with one arm spine and following tentacle pores with two arm spines. Inner arm spines longer than outer arm spines. On middle and distal part of arm, outer arm spines three-fourths as long as inner spines (Fig. 9F). Three arm spines occurred once in two of the five arms. Lateral arm plates not projecting on arms.

***Color*.** Bright pink in situ, entirely white in alcohol (Fig. 8B, C).

***Ossiclemorphology*.** Vertebrae articulation streptospondylous, wider than long in proximal segments (Fig. 10A, B), longer than wide in distal segments (Fig. 11A, B). Oral side of each vertebra with longitudinal groove along midline, deeply depressed, and no oral bridge (Figs 10C, 11C). Pair of podial basins on oral side moderate in size (Figs 10C, 11C). Aboral side of each arm vertebra with longitudinal aboral groove,moderately depressed (Figs 10D, 11D). Lateral furrow of vertebrae declining obliquely from aboral to oral side (Figs 10E, F, 11E, F). Lateral arm plates crescent-shaped, each associated with one or two arm spines. Spine articulations with separated nerve and muscle openings, bulging outwards (Fig. 12A, C). A ridge on inner side of lateral arm plate (Fig. 12D). Arm spines cylindrical, never hooked, bearing fine thorns at apex throughout arms (Fig. 12E, F).

###### Remarks.

*Ophiocreasabyssicola* was first described by Lyman (1879). Okanishi and Fujita (2014) transferred O. *abyssicola* to the genus *Astrodia* and redescribed it. This specimen (RSIO68002) was identical to *Astrodiaabyssicola* by having 0~2 arm spines, rather short genital slits and crescent-shaped lateral arm plates. However, this specimen lacks external ossicles on the disc and arms, which is different from previous descriptions of *Astrodiaabyssicola* by Okanishi and Fujita (2014) as having plate-shaped external ossicles on the periphery. Nevertheless, the genetic distance of COI and 16S (2.9% and 1.9%) between the new collected specimen and *A.abyssicola* are too small to justify two different species. Therefore, this specimen was identified as *A.abyssicola*, thus the external ossicles on the aboral surface of the disc could be plate-shaped or absent in this species.

### ﻿Key morphological characters to the species of *Astrodia*

The key morphological characters among the five species from the genus *Astrodia* based on Okanishi and Fujita (2014) were revised in this study (Table 3). Three diagnostic characteristics were proposed by Okanishi and Fujita (2014) in their key for *Astrodia*: the length of the genital slits related to the height of the disc, external ossicles on the aboral disc surface, and shape and existence of projections of lateral arm plates. All three characteristics were useful to distinguish the new species from its congeners. The external ossicle, being absent in the *A.abyssicola* specimen examined in the present study but present and plate-shaped in the previous descriptions (Okanishi and Fujita 2014), might be an intraspecific variation. Additionally, we added two morphological characters, the number of arm spines and the shape of oral papillae, as key characters for interspecific discrimination of *Astrodia*. *Astrodiaabyssicola* is the only species that possesses no more than two arm spines along their arms, whereas the other four species possess up to three arm spines or occasionally four. Furthermore, oral papillae are indistinct or underdeveloped in *A.duospina* sp. nov., but are domed granule-shaped in the four known species. Thus, we consider the number of arm spines and the shape and existence of oral papillae important characteristics for interspecific discrimination within *Astrodia* (Table 3).

**Table 3.** Comparison of key morphological characters among species in the genus *Astrodia*.

Species	Arm spines	Genital slits	External ossicles	Lateral arm plates on middle to distal portion of arms	Oral papillae	Reference
***Astrodiaabyssicola* (Lyman, 1879)**	0–2	very short, ~1/5 (height of disc)	plate-shaped on periphery	shapes: crescent; projections: absent	domed granule-shaped	Lyman (1879), Okanishi and Fujita (2014), This study
***Astrodiaexcavata* (Lütken & Mortensen, 1899)**	0–3	large, ~2/3 (height of disc)	granule-shaped near radial shields and genital slits	shapes: bar-like; projections: present	domed granule-shaped	Lütken and Mortensen (1899), Okanishi and Fujita (2014)
***Astrodiaplana* (Lütken & Mortensen, 1899)**	0–3	short, ~1/4 (height of disc)	absent	shapes: oblong; projections: absent	domed granule-shaped	Lütken and Mortensen (1899), Döderlein (1927), Okanishi and Fujita (2014)
***Astrodiatenuispina* (Verrill, 1884)**	0–3, occasionally 4	short, ~1/2 (height of disc)	plate-shaped on periphery, granule-shaped in center	shapes: unknown; projections: absent	domed granule-shaped, small and short	Verrill (1884), Koehler (1906), Koehler (1922), Baker (1980), Gage et al. (1983), Manso (2010), Okanishi and Fujita (2014)
***Astrodiaduospina* sp. nov.**	0–2, occasionally 3	short, ~1/4 (height of disc)	plate-shaped on periphery and in center	shapes: crescent; projections: absent	indistinct or underdeveloped	This study

### ﻿Molecular phylogenetic analysis

**Figure 13.**
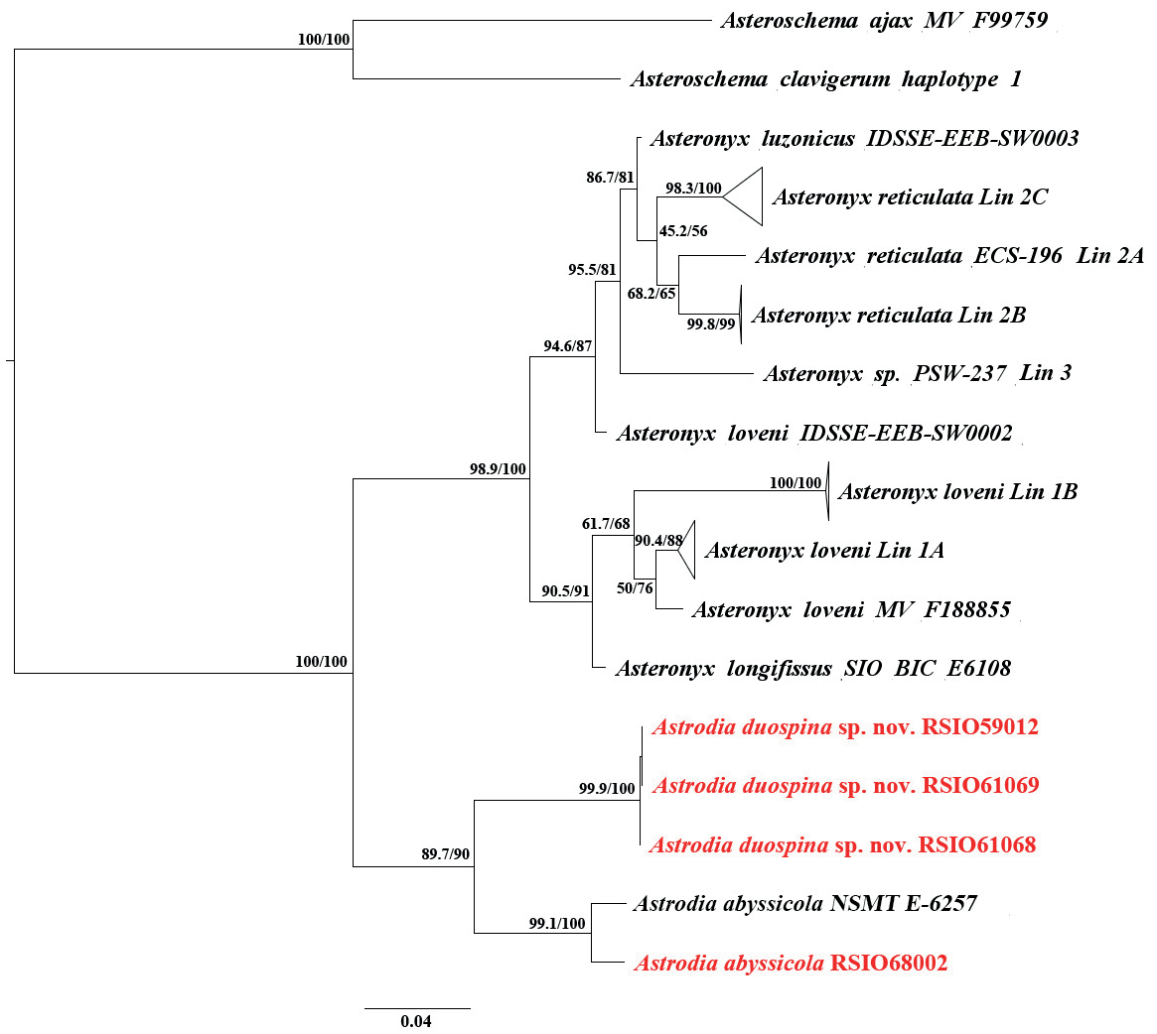
Maximum likelihood tree of the genus *Astrodia* based on concatenated sequences of COI and 16S (clades of Lin 1A, Lin 1B, Lin 2A, Lin 2B, Lin 2C and Lin 3 are from Okanishi et al. (2018), more detailed information about these clades showed in Suppl.materials. Values of each clade: SH-aLRT support (%) / ultrafast bootstrap support (%)).

Based on the COI (583~1511 bp) and 16S (431~539 bp) sequences, the phylogenetic relationship of the two genera, *Astrodia* and *Asteronyx*, was inferred. The ML tree based on the concatenated 16S and COI sequences suggested that both *Astrodia* and *Asteronyx* were monophyletic with high bootstrap values (Fig. 13, Suppl. material 1: Fig. S1). The ML tree based on COI sequences was consistent with the tree generated from two genes (Suppl. material 1: Fig. S2), while in the ML tree based on 16S sequences, *Astrodiaabyssicola* clustered with *Asteronyx*, with a low bootstrap value (Suppl. material 3: Fig. S3). Okanishi et al. (2018) suggested that the relationship of the two genera was unclear based on COI and 16S sequences. With newly sequenced DNA data added, our results indicated that the two genera are probably monophyletic. Additionally, the genetic distances of CO1 and 16S between *A.duospina* sp. nov. and *A.abyssicola* were 9.0% and 9.1%, respectively, supporting the morphological identification results. Molecular analysis also supported that the three specimens identified as *Astrodiaduospina* sp. nov. are the same species, and the specimen identified as *Astrodiaabyssicola* is closely related to the published sequence of this species with very small genetic distances (2.9% for COI and 1.9% for 16S) that fall into the intra-species genetic distance of Euryalida (Okanishi et al. 2018; Nethupul et al. 2022).

## ﻿Conclusion

In this study, we described a new species of the genus *Astrodia* collected from seamounts in the West Pacific, and another species (*Astrodiaabyssicola*) was redescribed. Through comparing the five species of *Astrodia*, the tabular key of Okanishi and Fujita (2014) was revised and two additional key characteristics, the number of arm spines and the shape of the oral papillae, were identified for interspecific discrimination of *Astrodia*. Maximum likelihood trees supported our morphological results and suggested that both *Astrodia* and *Asteronyx* were monophyletic. This study provided both morphological and molecular information of the two *Astrodia* species, and the specimens reported further expanded the known geological distribution of the genus.

## Supplementary Material

XML Treatment for Astrodia duospina

XML Treatment for Astrodia abyssicola

## Supplementary materials

Supplementary material 1 Figure S1 Data type Image. Explanation note Maximum likelihood tree of the genus *Astrodia* and *Asteronyx* based on concatenated sequences of COI and 16S. https://binary.pensoft.net/file/752863This dataset is made available under the Open Database License (http://opendatacommons.org/licenses/odbl/1.0/). The Open Database License (ODbL) is a license agreement intended to allow users to freely share, modify, and use this Dataset while maintaining this same freedom for others, provided that the original source and author(s) are credited.Xiaojun Xie, Dongsheng Zhang

Supplementary material 2 Figure S2 Data type Image. Explanation note Maximum likelihood tree of the genus *Astrodia* and *Asteronyx* based on COI sequences. https://binary.pensoft.net/file/752864This dataset is made available under the Open Database License (http://opendatacommons.org/licenses/odbl/1.0/). The Open Database License (ODbL) is a license agreement intended to allow users to freely share, modify, and use this Dataset while maintaining this same freedom for others, provided that the original source and author(s) are credited.Xiaojun Xie, Dongsheng Zhang

Supplementary material 3 Figure S3 Data type Image. Explanation note Maximum likelihood tree of the genus *Astrodia* and *Asteronyx* based on 16S sequences. https://binary.pensoft.net/file/752865This dataset is made available under the Open Database License (http://opendatacommons.org/licenses/odbl/1.0/). The Open Database License (ODbL) is a license agreement intended to allow users to freely share, modify, and use this Dataset while maintaining this same freedom for others, provided that the original source and author(s) are credited.Xiaojun Xie, Dongsheng Zhang
