# Supplementary figures and images for: ﻿Description of two species of the genus Astrodia Verrill, 1899 (Ophiuroidea, Euryalida, Asteronychidae), including a new species from seamounts in the West Pacific
Source: Zookeys. 2022 Oct 4;1123:99–122. doi: 10.3897/zookeys.1123.87397 (PMC9836647; doi:10.3897/zookeys.1123.87397)

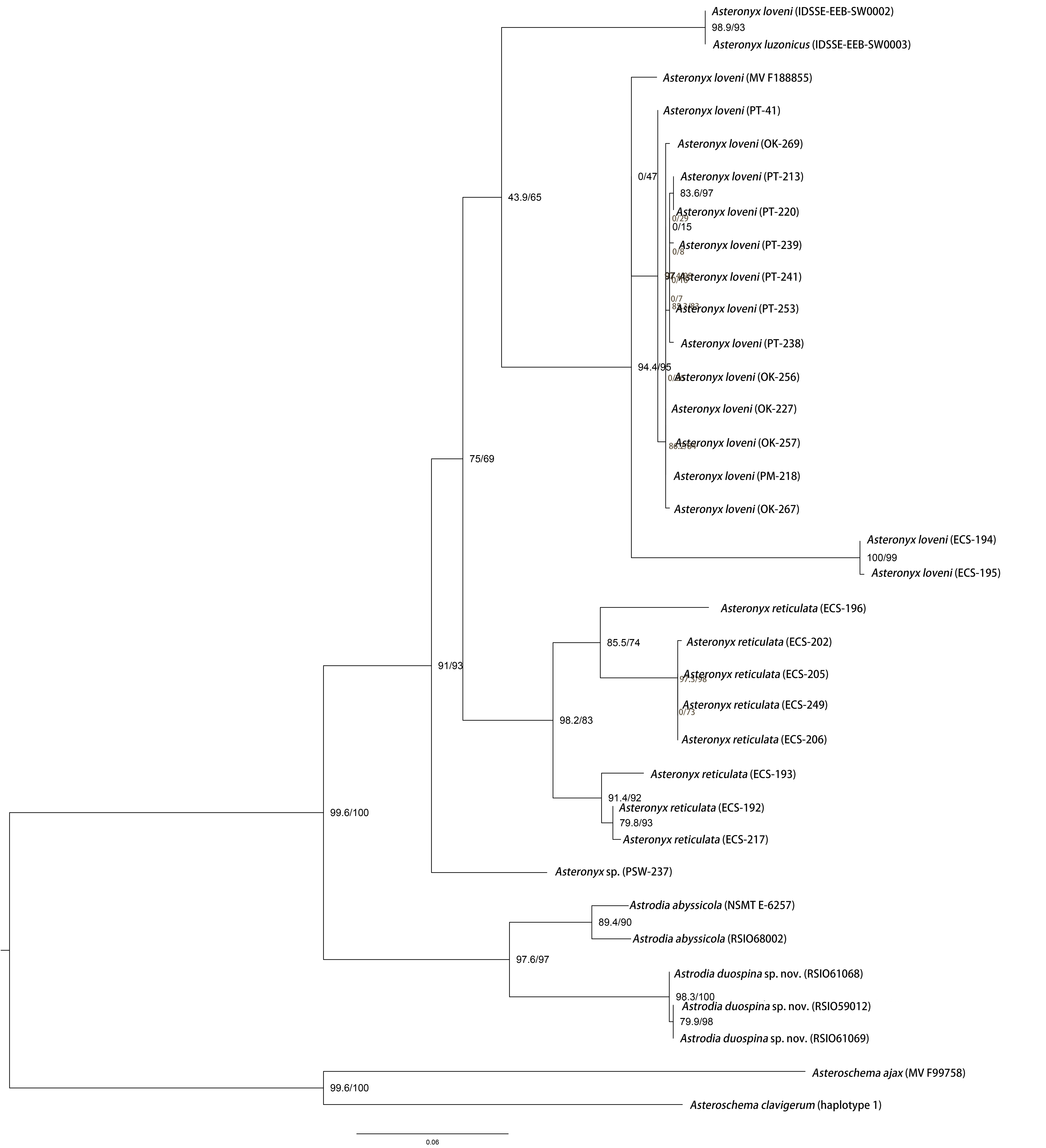

Supplement: Supplementary material 2 — Figure S2 [file zookeys-1123-099_article-87397__-s002.jpg]
